# Breastfeeding practices, timing of introduction of complementary beverages and foods and weight status in infants and toddlers participants of a WIC clinic in Puerto Rico

**DOI:** 10.1186/s40064-016-3154-9

**Published:** 2016-08-30

**Authors:** Olga E. Sinigaglia, Elaine M. Ríos, Maribel Campos, Beatriz Díaz, Cristina Palacios

**Affiliations:** 1 Nutrition Program, Department of Human Development, Graduate School of Public Health, Medical Sciences Campus, University of Puerto Rico, San Juan, PR 00936-5067 USA; 2 Undergraduate Department, School of Nursing, Medical Sciences Campus, University of Puerto Rico, San Juan, PR 00936-5067 USA; 3 Center for Clinical Research and Health Promotion, School of Dental Medicine, Medical Sciences Campus, University of Puerto Rico, San Juan, PR 00936-5067 USA

**Keywords:** Breastfeeding, Introduction of beverages and foods, Obesity, Infants, Hispanics

## Abstract

**Background:**

Breastfeeding is associated with lower rates of obesity; in addition, it is also associated with later introduction of beverages and foods; however, this has not been well studied among Hispanics. The objective was to assess breastfeeding practices and timing of introduction of beverages and solid foods in a sample of Hispanic infants and their association with weight status.

**Subject and methods:**

Cross-sectional study in 296 caregivers of infants and toddlers 0–24 months of age participants of a WIC clinic in Puerto Rico. Participants completed several questionnaires and anthropometrics were taken in infants and toddlers. Statistical analysis included correlations, comparison between groups and logistic regression.

**Results:**

A total of 189 participants older than 6 months completed the study. Most infants were breastfed immediately after birth (63.5 %), at the hospital (80.0 %), and at least once (92.3 %) but only 31 % were exclusively breastfed. Median duration of any breastfeeding was 5.0 months and exclusive breastfeeding was 0 months. Excessive weight was found in 22.8 %. Breastfeeding duration was positively associated with caregiver’s educational level and age of introduction of water, formula, juice and cow’s milk (*p* < 0.05). Exclusively breastfed infants were introduced water and formula at a later age compared to non-exclusively breastfed and never breastfed infants (*p* < 0.01). No significant associations were found between breastfeeding practices and duration or age of introduction of beverages and foods with weight status, even after adjusting for potential confounders.

**Conclusions:**

Breastfeeding duration was in general low. Water, formula and juice were introduced later in breastfed infants compared to non-exclusively breastfed or never breastfed infants. Breastfeeding practices or timing of introduction of beverages and solid foods were not significantly associated with weight status. Strategies to support mothers on continuing breastfeeding beyond the hospital and for longer periods are needed among WIC participants to benefit of the protective effect on childhood obesity.

## Background

Exclusive breastfeeding is recommended by many organizations during the first 6 months of life for healthy growth and development, including obesity prevention (American Pediatric Academy 2015; World Health Organization 2003). It has been estimated that for each month of exclusive breastfeeding, there is a reduction of 4 % in the risk of obesity later in life, with the lowest risk among those breastfed for up to 9 months of age (OR 0.68, 95 % CI 0.50, 0.91) (Harder et al. 2005).

Breastfeeding rates in the US have been steadily increasing over the years (Center for Disease Control and Prevention 2014). In 2014, the Center for Disease Control and Prevention reported that 79.2 % initiated breastfeeding, 40.7 % breastfeed exclusively for 3 months and 18.8 % for 6 months (Centers for Disease Control and Prevention 2016). However, breastfeeding initiation rate among participants of the Women, infant and children (WIC) program is lower (66 %), with only 12.5 % exclusive breastfeeding for 6 months in 2013 (Division SFP 2013). Breastfeeding duration is also associated with later introduction of complementary feeding (Baker et al. 2004; Giovannini et al. 2004; Schack-Nielsen et al. 2010). Furthermore, early introduction of complementary foods (<4 months) is also associated with excessive infant weight (Gaffney et al. 2012; Grote et al. 2012).

There is scarce data on infants and toddlers’ breastfeeding patterns among Hispanics/Latinos, a group with large health disparities in the US. Data from a nationally representative sample of WIC clinics showed the highest breastfeeding initiation rate among Hispanics/Latinos (84 % compared to non-Hispanic Whites, 53 %) (Sutherland et al. 2012); however, Hispanics/Latinos are less likely to practice exclusive breastfeeding than other groups (WIC Program 2002). Also, there is lack of information about the association between breastfeeding, introduction of complementary foods and weight status among this group. In particular, Puerto Rico has the highest obesity rates in children (Elías-Boneta et al. 2015) and in adults (Centers for Disease Control and Prevention 2013) compared to other states and territories in the US. Compared to other Hispanic/Latino groups in the US, Puerto Rican adults have the highest rates of obesity and cardiovascular disease risk (Daviglus et al. 2012). Understanding how early dietary practices relates to weight status in infants may help develop programs to prevent obesity later in life. Therefore, the objective of the present study was to assess breastfeeding practices and timing of introduction of beverages and solid foods in a sample of Hispanic infants’ participants of the WIC program in Puerto Rico and to assess the association of these variables with weight status. This information could help design proper interventions for improving dietary patterns and preventing obesity at early ages in this at risk population.

## Methods

### Study and sample design

This was a cross-sectional study in caregivers of infants and toddlers 0–24 months of age participants of a WIC clinic in Puerto Rico. Caregivers completed two questionnaires (socio-demographics and breastfeeding practices) and anthropometrics were taken in the infants and toddlers. This study was approved by the Institutional Review Board of the University of Puerto Rico Medical Science Campus and caregivers provided written consent before participating in the study.

### Participants

We recruited a non-probabilistic convenient sample of caregivers 21 years or older of infants and toddler aged 0–24 months. Recruitment occurred every day in the WIC clinic of the Municipality of Trujillo Alto, which is within the Metropolitan area of San Juan in Puerto Rico, during a 3-month period (November 2014–February 2015). This clinic is the only clinic in this municipality, which allowed recruiting all active infant participants of that clinic (n = 476), as caregivers of infants and toddlers attend the clinic on average once per month for their appointments or to pick-up their monthly checks. A total of 296 participants were recruited during this 3-month period. Approximately 10 caregivers refused to participate and an additional 15 were younger than 21 years. In addition, infants or toddlers with any serious health condition that could alter normal feeding practices were excluded.

At the time of recruitment, participants completed the two paper questionnaires, which took about 20 min to complete, and anthropometrics were taken in the infant as explained below.

### Socio-demographics

Caregivers completed a short self-reported questionnaire with questions on parent/caregivers’ age, gender, education and household size, questions on infants’ birth weight, age and gender and questions about pregnancy complications and weeks of gestation.

### Breastfeeding practices questionnaire

This questionnaire was based on questions adapted from the Southampton Women’s Survey (Fein et al. 2008; Marriott et al. 2009) and Centers for Disease Control and Prevention (CDC) Infant Feeding Practices study (Fein et al. 2008). The questions assess initiation and duration of breastfeeding (Fein et al. 2008), duration of exclusive breastfeeding (Marriott et al. 2007) and timing of introduction of water and complementary fluids such as formula (Marriott et al. 2007). Other added questions included breastfeeding initiation (immediately after birth or any time during the hospital), breastfeeding practices (exclusive or partial), duration of any type of breastfeeding, and timing of introduction of juice, solid foods and cow’s milk. There were 23 questions and it was administered via interview by trained research personnel.

### Anthropometrics measurements

Weight and recumbent length were measured by trained research personnel. For this, infants were weighed with light clothing, no shoes or socks and clean diaper. Weight was taken in duplicates in pounds using a manual calibrated scale (Detecto, MO). The average of both measurements was used, which was converted to kg. Recumbent length was measured in duplicates in cm using an infantometer (Perspective Enterprices, MI) and the average of both measurements was used. Weight status was calculated using weight-for-length growth charts from the World Health Organization by age and gender. Weight was classified as: underweight (<5th), healthy weight (5th–89th) and excessive weight (≥90th). For premature infants (n = 34, 11.7 %), gestational age was adjusted first before calculating the weight-for-length percentile.

### Statistical analysis

For this analysis, infants younger than 6 months were excluded to assess duration of breastfeeding for at least 6 months on weight status. Breastfeeding was analyzed as breastfed immediately after birth, at the hospital, at least once, and exclusively. In addition, breastfeeding duration was analyzed separately for any breastfeeding duration and exclusively breastfeeding duration.

For descriptive statistics, median (25th and 75th percentiles) were computed for continuous variables and frequencies for categorical values. To associate breastfeeding practices (start of breastfeeding, exclusive or non-exclusive breastfeeding and duration) with socio-demographics (age of mother, education, number of children), timing of introduction of beverages (water, formula, juice and cow’s milk) and solid foods, and infant weight status, we used Spearman correlations and logistic regression, unadjusted, adjusted for infant’s age and also adjusted for infant’s sex and caregiver’s age and educational level. To compare timing of introduction of beverages and solid foods by feeding types (never breastfed, non-exclusively breast-fed and exclusively breastfed, Kruskal–Wallis was computed. The data analysis was performed using the Statistical Package for the Social Sciences (SPSS), version 21.0. Statistical significance was set at *p* < 0.05.

## Results

A total of 296 infants/toddlers were recruited for the study. From the total recruited, we excluded infants younger than 6 months (n = 102) to allow to capture breastfeeding practices during the 6 months. Also, we excluded those with incomplete data (n = 4) and one for being older than 24 months. Therefore, the total sample included in this analysis was 189 subjects. Most of the caregivers were mothers (95.8 %) and had an educational level of greater than high school (62.0 %) and median caregivers’ age was 27.0 years (Table 1). Most infants/toddlers were boys (54.5 %) and median age was 12 months. Excessive weight was found in 22.8 % of infants and toddlers.

**Table 1:** Socio-demographics characteristics of the sample (n = 189)

Socio-demographic variables	N	% or Median (25th, 75th percentiles)
*Caregivers’ characteristics*		
Relation to infant		
Mother	181	95.8 %
Father	3	1.6 %
Other (grandparent, aunt/uncle)	5	2.6 %
Age (years)		27.0 (24.0, 32.0)
Education^a^		
≤High school	71	38.0 %
>High school	116	62.0 %
Number of children		2.0 (1.0–2.0)
*Infants’ characteristics*		
Gender		
Girl	86	45.5 %
Boy	103	54.5 %
Age (months)		12.0 (9.0,18.0)
Weight-for-length percentile		
Underweight (<5th)	5	2.6 %
Healthy weight (5–89th)	141	74.6 %
Excessive weight (≥90th)	43	22.8 %

^a^Missing data for 2 participants

Most infants were breastfed immediately after birth (63.5 %), at the hospital (80.0 %), and at least once (92.3 %) (Table 2). A total of 49 % were breastfed (non-exclusively) for 6 months while only 24 % were exclusively breastfed for 6 months. In terms of timing of introduction of other beverages and foods, water was introduced at a median age of 3.0 months, formula at 0.1 months, solid foods at 6.0 months, juice at 6.0 months and cow’s milk at 12.0 months.

**Table 2:** Breastfeeding practices and timing of introduction of complementary beverages and foods in the sample (n = 189)

Practices	% or Median (25th, 75th percentiles)
Breastfeeding immediately after birth	63.5 %
Breastfeeding at hospital	80.0 %
Breastfeeding at least once	92.6 %
Breastfed for 3 months (non-exclusively)	69 %
Breastfed for 6 months (non-exclusively)	49 %
Exclusive breastfeeding for 3 months	30 %
Exclusive breastfeeding for 6 months	24 %
Any breastfeeding duration (months)	5.0 (2.0, 10.0)
Exclusively breastfeeding duration (months)	0 (0, 5.0)
Age of introduction of water (months)	3.0 (2.0, 6.0)
Age of introduction of formula (months)	0.1 (0, 3.0)
Age of introduction of solid foods (months)	6.0 (6.0, 8.0)
Age of introduction of juice (months)	6.0 (5.0,7.0)
Age of introduction of cow’s milk (months)	12.0 (11.0, 12.0)

Table 3 shows the correlation between breastfeeding duration, socio-demographic characteristics and timing of introduction of water, formula, juice, cow’s milk and solid foods. Breastfeeding duration was positively associated with caregiver’s educational level (*p* < 0.05). Age of introduction of water, formula, juice and cow’s milk was directly related to duration of any breastfeeding (*p* < 0.05). Similarly, age of introduction of water, formula, and juice was directly related to duration of exclusive breastfeeding (*p* < 0.05).

**Table 3:** Spearman correlation between breastfeeding duration, socio-demographic characteristics and timing of introduction of complementary beverages and foods

Variables	Duration of any breastfeeding R (*p* value)	Duration of exclusive breastfeeding R (*p* value)
Age of parents (years)	0.07 (*p* = 0.14)	0.10 (*p* = 0.10)
Number of children (count)	0.06 (*p* = 0.20)	0.04 (*p* = 0.31)
Education (years)	0.28 (*p* < 0.05)*	0.17 (*p* < 0.05)*
Age of introduction of water (months)	0.40 (*p* < 0.05)*	0.27 (*p* < 0.05)*
Age of introduction of formula (months)	0.37 (*p* < 0.05)*	0.50 (*p* < 0.05)*
Age of introduction of solid foods (months)	0.10 (*p* = 0.11)	0.10 (*p* = 0.11)
Age of introduction of juice (months)	0.27 (*p* < 0.05)*	0.20 (*p* < 0.05)*
Age of introduction of cow’s milk (months)	0.24 (*p* < 0.05)*	0.05 (*p* = 0.31)

Exclusively breastfed infants were introduced water (5.0 months) and formula (2.0 months) at a later age compared to those who were not exclusively breastfed (3.0 and 0.1 months, respectively) and those that were never breastfed (water 2.0 and formula 0 months; *p* < 0.01) (Table 4). Juice was introduced at 6.0 months in those breastfed (non-exclusively or exclusively) compared to 5.0 months in those never breastfed (*p* < 0.01). Solid foods were introduced at 6 months and cow’s milk at around 11–12 months in all groups (*p* > 0.05).

**Table 4:** Timing of introduction of complementary beverages and foods by type of feeding

Variables	Median (25th, 75th percentiles)			*p* value^a^
	Never breastfed N = 21	Non-exclusive breastfed N = 109	Exclusive breastfed N = 59	
Age of introduction of water (months)	2.0 (0, 3.0)	3.0 (2.0, 6.0)	5.0 (2.0, 6.0)	0.001*
Age of introduction of formula (months)	0 (0, 0)	0.1 (0, 2.0)	2.0 (0, 5.0)	0.002*
Age of introduction of solid foods (months)	6.0 (5.5, 8.0)	6.0 (6.0, 7.0)	6.0 (6.0, 9.0)	0.60
Age of introduction of juice (months)	5.0 (4.0, 5.5)	6.0 (4.0, 6.3)	6.0 (5.0, 9.0)	0.006*
Age of introduction of cow’s milk (months)	11.0 (10.5, 12.0)	12.0 (12.0, 12.0)	12.0 (12.0, 12.0)	0.17

* *p* < 0.05

^a^Kruskal-Wallis

Table 5 shows the unadjusted and adjusted odds ratios from logistic regression examining the association between breastfeeding practices and weight status. Breastfeeding immediately after birth was associated with 0.54 (0.26, 1.11) lower odds of excessive weight after adjusting for infant’s age and sex and parent’s age and education. Similarly, breastfeeding at least once was associated with 0.46 (0.14, 1.50) lower odds of excessive weight in the multivariate model. Similar results were obtained for breastfeeding for 3 or 6 months (non-exclusively) and for 6 months exclusively. However, none of these associations reached the significant level.

**Table 5:** Unadjusted and adjusted odds ratios from logistic regression examining the association between breastfeeding practices and duration and weight status

Variable	OR (95th confidence intervals)		
	Crude	Adjusted for infant’s age	Multivariate^a^
Breastfed immediately after birth	0.58 (0.29, 1.16)	0.58 (0.30, 1.16)	0.54 (0.26, 1.11)
Breastfed at the hospital	0.94 (0.40, 2.17)	0.94 (0.40, 2.17)	0.90 (0.38, 2.14)
Breastfed at least once	0.50 (0.16, 1.58)	0.50 (0.16, 1.58)	0.46 (0.14, 1.50)
Breastfed for 3 months (non-exclusively)	0.62 (0.30,1.26)	0.62 (0.30, 1.26)	0.55 (0.27, 1.16)
Breastfed for 6 months (non-exclusively)	0.77 (0.39, 1.53)	0.77 (0.39, 1.53)	0.66 (0.32, 1.37)
Exclusive breastfeeding for 3 months	0.87 (0.41, 1.85)	0.87 (0.41, 1.85)	0.80 (0.37, 1.74)
Exclusive breastfeeding for 6 months	0.67 (0.29, 1.58)	0.67 (0.27, 1.65)	0.60 (0.25, 1.45)

^a^Adjusted for infant’s age and sex and parent’s age and education. Reference: healthy weight

Table 6 shows the unadjusted and adjusted odds ratios from logistic regression examining the association between timing of introduction of water, formula, juice, cow’s milk and solid foods with weight status. No significant associations were noted in any of the variables included.

**Table 6:** Unadjusted and adjusted odds ratios from logistic regression examining the association between timing of introduction of complementary beverages and foods and weight status

Variable	OR (95th confidence intervals)		
	Crude	Adjusted for infant’s age	Multivariate^a^
Age of introduction of water (months)	0.95 (0.83, 1.08)	0.95 (0.83, 1.08)	0.92 (0.79, 1.06)
Age of introduction of formula (months)	0.89 (0.75, 1.04)	0.88 (0.75, 1.04)	0.86 (0.73, 1.02)
Age of introduction of solid foods (months)	0.97 (0.83, 1.14)	0.95 (0.81, 1.12)	0.96 (0.81, 1.13)
Age of introduction of juice (months)	1.06 (0.92, 1.23)	1.07 (0.92, 1.24)	1.06 (0.91, 1.23)
Age of introduction of cow’s milk (months)	1.02 (0.82, 1.26)	1.01 (0.82, 1.25)	1.03 (0.82, 1.29)

^a^Adjusted for infant’s age and sex and parent’s age and education. Reference: healthy weight

## Discussion

In this study we assessed breastfeeding practices, timing of introduction of other beverages and foods and weight status in infants and toddlers participants of a WIC clinic in Puerto Rico. Excessive weight was found in 22.8 % of the infants, which is higher when we compared with other WIC studies (Sekhobo et al. 2010). Breastfeeding initiation rate was high (92.6 %), and this rate is higher than the Healthy People 2020 goal (80 %) (U.S. Department of Health and Human Services 2010). However, only 49 and 31 % were breastfed for 6 months, non-exclusively or exclusively, respectively. This is similar to reports from the CDC in Hispanics (Center for Disease Control and Prevention 2014; Centers for Disease Control and Prevention 2016).

According with our data, higher caregiver’s education was correlated with greater duration of any breastfeeding or exclusive breastfeeding. Similarly, the Infants Feeding Practices II found that infants from mothers with a higher educational level breastfed longer (Grummer-Strawn et al. 2008). However, parent’s age or number of children in the family was not associated with breastfeeding duration, contrary to other studies (Sutherland et al. 2012). Also, we found that breastfeeding was associated with later introduction of water, formula, juice and cow’s milk but not of solid foods. When compared by type of feeding, exclusively breastfed infants are introduced water, formula and juice later than non-exclusively breastfed or never breastfed infants. Independently of type of feeding, juices were introduced considerably earlier in the sample compared with WIC complementary guidelines, which recommend starting juice at 12 months or later (WIC Program 2002). However, introduction of solids and cow’s milk was similar and according to recommendations (WIC Program 2002). Others have found that the introduction of solid foods before 4–6 months is associated with discontinuation of breastfeeding (Grummer-Strawn et al. 2008; González-Cossío et al. 2006).

Breastfeeding practices and longer duration of breastfeeding was associated with lower odds of excessive weight gain but these associations were non-significant. We expected to find significant associations between breastfeeding and weight status as other studies have found (Koletzko et al. 2009). Breastfeeding for more than 4 months has been found to be protective of overweight (OR 0.81; 95 % Cl 0.71–0.92) but it was not protective if breastfeeding was shorter than 4 months (Grube et al. 2015). It is important to note that most studies have shown the protective effect on weight later in childhood and not immediately (Harder et al. 2005). Therefore, it would be necessary to follow this cohort to examine this in the future. Also, duration of any or exclusive breastfeeding in our study was very low (5 and 0 months, respectively), which may not be enough to provide the benefits on reducing excessive weight in infants. In fact, a study among a 201 un-acculturated Latino mothers from San Francisco found a lower risk of obesity with breastfeeding for 12 months or more (OR 0.39, 95 % CI 0.02–0.93) (Verstraete et al. 2014).

Duration of breastfeeding may be an independent predictor of weight status acting through the delay on the introduction of other foods and beverages, such as juice in our study, as recommended for preventing childhood obesity. However, we did not find a significant association between timing of introduction of water, formula, juice, cow’s milk or solid foods with weight status. Therefore, more studies are needed in Hispanics to identify other characteristics or practices associated with early infant excessive weight. Currently, most studies have been conducted among non-Hispanic whites or Blacks, with very few studies done among Hispanic/Latino infants. This group may have different characteristics that could modify the association between breastfeeding and weight (Grummer-Strawn and Mei 2004).

Our study has some strengths and limitations worth acknowledging. The cross-sectional design of the study does not allow us to infer causality. Longitudinal studies are needed with this population to determine the protective effect of long-term breastfeeding and timing of introduction of water, formula, juice, cow’s milk and solid foods later in childhood. In addition, we did not assess caregivers weight status, an important variable that should be included in future studies. A strength of this study is that it focuses on Puerto Ricans, a population with well-documented health disparities in diet-related chronic diseases but also with lack of studies to help determine the main risk factors. In this study we used trained interviewers, calibrated equipment’s and standardized procedures to conduct all the infant measurements. To recruit our sample, we visited a WIC clinic every day for 3 months to ensure that all current WIC participants could be included. In addition, we chose a WIC clinic that serves all the families of one municipality in Puerto Rico, to ensure a homogeneous and a more representative sample of that municipality.

In conclusion, we found in the present study that breastfeeding initiation rate was high but duration of any breastfeeding practices was rather low. Introduction of solid foods and cow’s milk was according to guidelines but juice was introduced much earlier. Breastfeeding was associated with later introduction of water, formula and juice but not of cow’s milk or solid foods. We did not find a significant association between breastfeeding or between the timing of introduction of water, formula, juice, cow’s milk and solid foods with weight status in this sample of Hispanic infants. Strategies to support mothers on continuing breastfeeding beyond the hospital and for longer periods are needed among WIC participants to benefit of the protective effect on childhood obesity. In addition, more emphasis should be given on the recommendation to delay introduction of juices at 12 months and beyond.
